# State of Knowledge About Thyroid Cancers in the Era of COVID-19—A Narrative Review

**DOI:** 10.3390/biomedicines12122829

**Published:** 2024-12-13

**Authors:** Agnieszka Bronowicka-Szydełko, Maciej Rabczyński, Ilias Dumas, Żanna Fiodorenko-Dumas, Beata Wojtczak, Łukasz Kotyra, Irena Kustrzeba-Wójcicka, Łukasz Lewandowski, Beata Ponikowska, Aleksandra Kuzan, Joanna Kluz, Andrzej Gamian, Katarzyna Madziarska

**Affiliations:** 1 Department of Medical Biochemistry, Wroclaw Medical University, 50-368 Wroclaw, Poland; agnieszka.bronowicka-szydelko@umw.edu.pl (A.B.-S.); kotyra.lukasz@gmail.com (Ł.K.); irena.kustrzeba-wojcicka@umw.edu.pl (I.K.-W.); lukasz.lewandowski@umw.edu.pl (Ł.L.); 2 Clinical Department of Diabetology, Hypertension and Internal Diseases, Wroclaw Medical University, 50-556 Wroclaw, Poland; maciej.rabczynski@umw.edu.pl (M.R.); joanna.kluz@umw.edu.pl (J.K.); katarzyna.madziarska@umw.edu.pl (K.M.); 3 Department of Clinical Physiotherapy and Rehabilitation, Wroclaw Medical University, 50-368 Wroclaw, Poland; zanna.fiodorenko-dumas@umw.edu.pl; 4 University Center for General and Oncological Surgery, Wroclaw Medical University, 50-368 Wroclaw, Poland; beata.wojtczak@umw.edu.pl; 5 Department of Physiology and Pathophysiology, Division of Physiology, Wroclaw Medical University, 50-368 Wroclaw, Poland; beata.ponikowska@umw.edu.pl; 6 Department of Preclinical Sciences, Pharmacology and Medical Diagnostics, Wroclaw University of Science and Technology, 51-377 Wroclaw, Poland; aleksandra.kuzan@pwr.edu.pl; 7 Hirszfeld Institute of Immunology and Experimantal Therapy, Polish Academy of Sciences, 53-114 Wroclaw, Poland; andrzej.gamian@hirszfeld.pl

**Keywords:** *ACE2*, *CLEC4M*, levothyroxine, SARS-CoV-2, thyroid cancers, *TMPRSS2*

## Abstract

Thyroid cancer (TC), due to its heterogeneous nature, remains a clinical challenge. Many factors can initiate the carcinogenesis process of various types of TC, which complicates diagnosis and treatment. The presented review gathers current information on specific types of TC, taking into account the effects of the COVID-19 pandemic. It is likely that COVID-19 has influenced and continues to influence the function of the thyroid gland. A high percentage of patients with COVID-19 showing simultaneous pathological changes in the thyroid suggests that SARS-CoV-2 may disrupt the function of this gland and initiate pro-oxidative mechanisms, inflammatory states, and autoimmune diseases, thereby promoting the formation of neoplastic changes. Furthermore, changes in the expression of the *ACE2*, *TMPRSS2*, *CLEC4M* and *DPP4* genes, observed in TC, also occur in COVID-19. Therefore, it is probable that the interaction of SARS-CoV-2 with thyroid cell receptors may initiate carcinogenesis in this gland. Additionally, some drugs used in TC therapy (e.g., levothyroxine) may increase the affinity of SARS-CoV-2 for cells, which could contribute to a more severe course of COVID-19 and the emergence of long-term symptoms (post-COVID-19). Moreover, the consequences of sanitary restrictions (limited access to medical services, reduction in endocrinological and oncological procedures) that took place in many countries during the COVID-19 pandemic may lead in the future to an increased number of missed diagnoses and the emergence of aggressive cancers.

## 1. Introduction

According to the World Health Organization, malignant thyroid cancers are divided into two main histological types [1]: (1) those derived from follicular cells (a group of about 95–97% of TC’s cases): primary thyroid lymphomas (PTL), malignant thyroid teratoma, primary thyroid sarcoma, metastasis of other tumors, tumors of follicular cells (papillary (PTC), follicular (FTC), poorly differentiated (PDTC), and anaplastic (ATC)); and (2) those derived from parafollicular, C cells—parafollicular cell neoplasms including medullary (MTC), squamous-cell thyroid carcinoma (SCTC), primary thyroid lymphomas (PTL), malignant thyroid teratoma, primary thyroid sarcoma (PTS), and metastasis of other tumors to thyroid glands, which, altogether, constitute approximately 5% of thyroid cancers [2].

Thyroid cancer (TC) remains as the most common form of endocrine carcinoma, constituting approximately 95% of endocrine cancers and 3.4% of all carcinomas diagnosed annually all over the world. Global statistics indicate that the evolution of thyroid cancer’s biology may influence both its incidence and mortality [3].

It is hypothesized that the incidence rate of TCs and their related mortality are the results of the evolution of TC biology. Thyroid cancer is generated through a multi-step process of carcinogenesis, in which the genome of thyrocytes is damaged, accelerating the tumor’s ability to invade the surrounding tissues or metastasize to distant organs [4]. Initiation of the process of carcinogenesis in the thyroid gland can be caused by one or many co-existing genetic mutations, chromosomal rearrangements, and changes in the tumor environment caused by factors including oxidative, nitrosyl, and carbonyl stress, glycation, and viruses, one of these being Severe Acute Respiratory Syndrome Coronavirus 2 (SARS-CoV-2) [5,6]. Mutations of particular genes are not selective for a given type of thyroid cancer, and, moreover, they may also be observed in cancers of other organs. External factors activate intracellular signaling pathways (such as PI3K/AKT/NF-kB, p21/MEK/MPAK or JAK/STAT, RAS/ERK/p53), inducing the synthesis of interleukins and cytokines, which influence metastasis, angiogenesis, and proliferation of cancer. Mutated gene sites are the most common targets of drug action in TC therapy, as they inhibit the replication of TC cells. It has been observed that many of these drugs, in patients with TC and infection caused by Coronavirus Disease 2019 (COVID-19), also exhibit antiviral effects [7]. These effects may occur through various molecular mechanisms, not limited to the inhibition of SARS-CoV-2 replication. Although the phenomenon is discussed in detail in the second part of this review, the roles of specific oncogenes on the development of individual TCs are featured in the table below (Table 1).

The current paper is structured in two parts. The first includes a description of thyroid cancers, mainly in the context of histology, as well as some data on their incidence, associated genetic alterations, and diagnosis. In the second part, information on the impact of COVID-19 on TCs was collected and an attempt was made to answer the following questions: ‘Can SARS-CoV-2 lead to TC?’, ‘What is the prognosis of patients with TC who contracted SARS-CoV-2?’, and ‘How do drugs used in TCs affect the course of COVID-19?’.

## 2. Description of TCs

### 2.1. Papillary Cancer

Papillary thyroid carcinoma (PTC) is the most frequent among TCs (70–90%) [36]. There are a few risk factors involved in the development of PTC, including genetic changes, growth factors, and exposure to ionizing radiation. The most common genetic changes occurring in PTC are the following mutations: *BRAF V600E* (encodes serine-threonine kinase of B-Raf proteins engaged in sending signals inside cells and in managing cell growth) [37], N-Ras, H-Ras, K-Ras (proteins of cell cycle), and *EIF1AX* (associated with eukaryotic factor of chromosome X translation 1A initiation) [38]. Relocations within RET (receptor for tyrosine kinase) are a common phenomenon occurring in PTC, but observations show that these changes differ between particular PTCs and may occur in majority, in a small fraction, or in a few cancerous cells only [39], which stems from the polyclonal origin of the tumor. Studies on panels including eight genes (*BRAF*, *KRAS*, *HRAS*, *NRAS*, *EGFR*, *PIK3CA*, *KIT*, and *PDGFRA*) connected to TCs did not indicate presence of molecular connotations between analyzed tumor areas despite the fact that these areas were connected in terms of etiology and morphology (biological material samples that were analyzed were taken from 500 patients) [40,41]. The search for somatic variants of single nucleotide, insertions, deletions, genes’ fusions, and changes within the number of somatic copies allows scientists to elaborate the genomic scoring system *BRAF V600E-RAS* (BRS) [10], which allows us to identify PTC in 98.8% of cases.

In patients diagnosed as PTC-positive, medicine observes a precise correlation between survivability and age, tumor size, presence of distant metastases, and lymph node lesions [40]. PTC is characterized by slow growth, may develop metastases within thyroid flesh and in lymph nodes, and remains iodine-catching [42]. It is possible to distinguish some types of papillary cancer considered the following: size (papillary microcancer—a small, well-differentiated PTC that is 1 cm or less in size), structure (papillary follicular cancer, macrofillicular cancer, cribriform cancer, homogeneous cancer, micropapillary cancer—a variant of PTC containing very small papillary structures less than 1 mm in size), character of tumor borderline, presence of additional tumor components (papillary cancer with central islet-like component, papillary cancer with spindle and gigantocellularis cancer, papillary cancer with squamous cell carcinoma, papillary cancer with mucous-epidermal cancer), and features of tumor stroma. Often the classic variant (microscopicly homogeneous) of cancer is accompanied by the follicular variant. Aggressive variants of PTC, according to the newest WHO classification, include the following: columnar tumor, tall cell tumor, solid tumor, and the so called hobnail type (its papillary and micropapillary structures contain cells with eosinophil cytoplasm and apically located nucleus with distinguishable nucleolus, confused with mycosis of the thyroid gland) [43,44,45].

More than 50% of cases of the disease are accompanied by the presence of polyps [43]. However, diagnosis and treatment of PTC is problematic [46]; for example, patients with benign tubercle of the thyroid gland with papillary hyperplasia (BTN-PH) may be incorrectly cytologically interpreted as suffering from PTC and, as a result, exposed to excessive treatment such as lobectomy or total thyroidectomy [47]. Although PTC has a generally good prognosis, a fraction of PTC patients relapse and develop metastases. A minority of PTC is aggressive, with rapid progression and a poor prognosis [48].

### 2.2. Follicular Thyroid Carcinoma

Follicular thyroid carcinoma (FTC) is the second (after PTC) most common TC (10–15%). It is characterized by slow growth, may release metastases to bones and lungs, and is iodine-catching. Usually, FTC are more aggressive, show a more advanced stage at the moment of diagnosis, are less sensitive to traditional therapy, and have a mortality rate higher than in PTC [49]. However, aggressiveness is associated with the tumor subtype, as well as the presence of distant metastases, tumor size, and extrathyroidal extension. There are more aggressive subtypes of PTC (e.g., tall cell, columnar cell variant, diffuse sclerosing variant, solid/trabecular variant, etc.), as well as widely invasive FTC (in contrast to the classical PTC variant, follicular PTC variant, and minimally invasive FTC, which generally have a good prognosis) [50]. FTC most frequently occurs in women at the age of 40–60 years. The survival rate for this cancer is high, equaling 92%. FTC and PTC show genetic similarity too—both cancers show somatic mutations RAS and rearrangements in PAX8/PPAR [51]. Moreover, in FTC, some more mutations in genes have been observed, although they are not so frequent, namely *PI3CA*, *PTEN*, *DICER1*, *EZH1*, and *SPOP* [52], some of which are engaged in the PI3K/PTEN/AKT pathway, which has been found to be a key signaling pathway in FTC development [53].

Diagnosis stated on the basis of USG with aimed biopsy is difficult [54]. In case of FTCs in cytological assessment, it is most difficult to differentiate benign lesions (adenoma) and malignant ones [55]. The correct diagnosis is possible through surgical removal of the lesion and histopathological examination. The only sure sign of malignancy in histopathological examination is invasion to vessels and tumor capsule [54]. FTC is usually characterized by a well-formed capsule with blood vessels [51], and capsule invasion is an indicator of vessel invasion, which has diagnostic implications.

### 2.3. Poorly Differentiated Thyroid Carcinoma

Poorly differentiated thyroid carcinoma (PDTC) is a malignant neoplasm and constitutes between 2% and 5% of all TCs. It manifests itself most often in people over 60 years of age, more often in men. It has a poor prognosis: the relapse-free time is shorter than 12 months, the 5-year overall survival is approximately 72%, and approximately 54% of patients die within 10 years [8]. In PDTC, mutation is most frequently observed in gene *TP53* [56]; on the other hand, the occurrence of changes in *RET*, *PTC*, *PAX8*, and *PPR* genes is rare, which indicates that these mutations are not connected to differentiation of cells [57]. Moreover, in PDTC, mutations in *BRAF* and *RAS* genes (connected to metastases probability) are observed [57].

### 2.4. Anaplastic Thyroid Carcinoma

Anaplastic thyroid carcinoma (ATC) is the most heterogenous of all TC subtypes. ATC is an aggressive, malignant tumor, giving fast metastases to other tissues (ATC tumors are widely invasive and grow into thyroid flesh, surrounding soft tissues, and neck structures). Early spread of the tumor results in distant metastases in 20–50% of patients, and in 90%, in invasion into neighboring tissues [58]. Although ATC constitutes less than 2% of all TCs, it is responsible for about 50% of the mortality rate among TCs [59]. In ATC, heterogenous genetic changes appear with domination of effectors of protein kinase activated by mitogens and domination of genes included in the signaling pathway PI3K, β-catenin [60]. In ATC, mutations of genes *RAS* and *BRAF* often occur. A characteristic factor of ATC is an increase in the number of copies of *PIK3CA*, which often coexists with mutations of the *BRAF* gene [61]. Mutations in *TP53* (in about 70% of ATC cases) often occur, so detection of *TP53* mutation could be connected to the presence of subclones representing features specific to ATC development alone [62]. Moreover, conducting immunohistochemistry research using monoclonal antibody MRQ-50 showed that expression of *PAX* takes place in 50% of ATC cases. These results contradict the belief that expression of this gene occurs in 80% of ATC cases and that the protein of PAX8 is the most sensitive marker, which was suggested by the results of research using monoclonal antibody anti-PAX8 [63].

Diagnosis of ATC may be complicated because it can show a morphological overlap with other cancers of anaplastic morphology. ATC tumors are similar to fibrosarcoma, undifferentiated pleomorphic sarcoma, single fibrous tumor, angiosarcoma, and rhabdomyosarcoma. ATC is characterized by quick growth, may give early metastases via the bloodstream and lymphatic system, is not iodine-catching, and prognosticates badly.

ATC is difficult to treat and occurs most frequently in the elderly. ATC has a poor prognosis. Radical surgery is often impossible, and in many cases, radical radiotherapy or chemotherapy is not possible. In chemotherapy, doxorubicin monotherapy or multi-drug regimens are used. The method of treatment depends on the patient’s state of health.

### 2.5. Medullary Thyroid Carcinoma

Medullary thyroid carcinoma (MTC) is a rare cancer (incidence rate: 1 person/1 million/year [64]); more frequent in women [65], it constitutes 5–10% of all thyroid tumors [66]. It is responsible for 13.4% of all deaths due to cancer of this gland [65]. The probability rate of this cancer in accidentally detected tumors is 0.6% [67]. In 75% of patients, it is non-hereditary, and in the remaining 25%, it is genetic, being a part of multiple endocrine neoplasia part 2 (MEN2) [68]. It may occur in children, constituting 10% of all TCs, and almost every case is connected to MEN2 [69]. MTC is a cancer deriving from C cells, which physiologically constitute about 0.1% of the thyroid gland mass and are a main source of calcitonin in the body [70]. In the non-hereditary type, in 80–90% of cases, it has unifocal character, and in MEN2, it is multifocal [71]. MTC may occur sporadically or can accompany congenital MEN2 (20–30% of cases). Development of MTC may be caused by mutation in *RET* or *RAS* (mutations in *RET* and *RAS* contradict each other) [72]. Analysis of original tumors and metastases showed that about 80% of sporadic cases of MTC included at least 1 subpopulation of cancerous cells with mutations in codon 918 in the protooncogene *RET*, and the frequency of this mutation depended on the tumor region. Mutations of *RET* genes may arise during clonal evolution or may be connected with presence of other clones presenting this mutation.

### 2.6. Other Malignant TCs

Thyroid lymphoma is a cancer of the immune system. It appears as a result of the proliferation of B lymphocytes. Thyroid lymphoma is a relatively rare disease which remains relatively difficult to diagnose. Suspicion of thyroid lymphoma is raised by a rapidly growing mass in the neck accompanied by Hashimoto inflammation. In ultrasonography, this tumor presents itself as a huge, unilateral mass that is hypoechogenic and invading neighboring soft tissues. The first diagnostic test accelerating the final diagnosis is biopsy, which reduces the patient’s burden of additional tests and interventions [73]; nevertheless, reaching a final diagnosis may be late if an insufficient amount of biopsy material is taken for immunohistochemical analysis. Treatment of lymphoma depends on its stage and location. Low-grade lymphomas are treated with radiotherapy and high-grade lymphomas with chemotherapy. Monoclonal antibodies are also used. The need for surgical removal of the entire thyroid is rare.

Thyroid sarcoma occurs rarely, usually in young adults, and 4 times more frequently in men than women.

Squamous cell carcinoma is a rare cancer (less than 1% of all malignant TCs) and highly malignant (the average survival rate of 9–12 months) [74]; it is most frequent in people over 63 and twice as frequent in women than in men [75].

Giant thyroid tumors are rare neoplasms characterized by exceptionally large sizes (their diameter exceeds 10 cm). Some can even reach 20 cm or more. These tumors can result from various types of thyroid cancer, including papillary thyroid carcinoma (PTC) and follicular carcinomas [76].

## 3. The Effect of COVID-19 on the Thyroid Gland

### 3.1. Impact of the COVID-19 Pandemic on Mortality Due to TC

In many countries, the strict sanitary measures implemented during the COVID-19 pandemic (enforcing minimized in-person contact in healthcare facilities) led to the development of telemedicine, which proved sufficient for certain cases involving patients with differentiated thyroid carcinoma (DTC) and medullary thyroid carcinoma (MTC). However, direct contact was necessary for symptomatic patients, new patients, those enrolled in clinical programs, patients with DTC or MTC showing incomplete therapeutic responses, and individuals facing difficulties using technology [77]. Legal regulations concerning sanitary measures and healthcare operations varied across countries, but a general trend emerged: there was a reduction in the number of surgical procedures and an increase in endocrinology and oncology consultations conducted via telemedicine. In Italy, during the so-called “COVID-19 waves”, a slight decrease in thyroidectomy procedures for thyroid cancer was observed, which resolved as SARS-CoV-2 infection rates declined [78]. A study spanning China, South Korea, Iran, and Italy highlighted reductions in early stage TC treatments, surgery times, and length of hospitalization [79]. At the start of the pandemic, outpatient visits in China dropped by 93.3%, with no new TC diagnoses and a complete halt to surgical procedures. Although surgeries resumed in February 2020, they were limited to the most urgent cases [79]. However, pandemic-period statistics did not show an increased rate of aggressive cancers. However, this aspect requires ongoing monitoring, as delays in screening and planned diagnostics could lead to a future rise in undiagnosed cases and aggressive tumors [78]. Alongside reduced surgeries, some services were also minimized. For example, in Europe, during the most challenging months of the pandemic, a significant decrease in isotope-based diagnostics (58.4% for thyroid scans) and therapies (41.8% reduction) complicated radioactive iodine (I-131) treatment [80]. The pandemic also caused a 99.7% reduction in thyroid fine-needle aspiration biopsies (FNABs) during its early months [79]. Delaying TC surgeries by three months was estimated to increase 10-year mortality risk by approximately 3% [81]. Additionally, TC patients awaiting thyroidectomy often experienced stress and anxiety, negatively affecting their mental and physical health [82].

Analyses of death registries for thyroid cancer (TC) conducted so far in various parts of the world, such as studies of the Wonder database (Wide-Ranging Online Data for Epidemiologic Research) and records from the U.S. Centers for Disease Control and Prevention (CDC) from 2018–2022, have not shown an increase in deaths among TC patients compared to the pre-pandemic period [83,84]. Moreover, no significant relationship was found between TC diagnosis, the therapy used, and the severity of COVID-19 [85]. It is therefore likely that timely TC treatment, with priority given to patients with advanced stages of the disease during the COVID-19 pandemic, helped maintain early TC mortality at levels comparable to the pre-pandemic period [83].

Interestingly, the diagnostic protocols for patients with severe COVID-19 (e.g., measuring inflammatory markers such as CRP and procalcitonin [PCT]) may, in some cases, have contributed to faster TC detection, particularly for medullary thyroid cancer (MTC). Studies have shown that elevated PCT levels, commonly tested in severe COVID-19 cases to confirm or rule out bacterial infections, do not necessarily stem from infection but could indicate thyroid cancer (most often MTC), as PCT is a precursor of calcitonin produced in the thyroid. This hypothesis is supported by case studies of COVID-19 patients from various parts of the world, where significantly elevated PCT levels were observed despite the normalization of inflammatory markers during hospitalization. In the UK, a case involved a 77-year-old woman who had undergone surgical removal of MTC in 1980 was described [86]. Her PCT levels remained consistently elevated (>100 ng/L) throughout hospitalization, despite normal CRP levels and white blood cell counts. Imaging diagnostics and a one-time calcitonin test revealed that the elevated PCT level was due not to bacterial infection but to PCT synthesis in the metastases of MTC, even though these metastases were stable. A similar diagnosis occurred in a 43-year-old woman who presented with moderate COVID-19 in April 2021. Despite clinical improvement and normalization of inflammatory markers, her PCT level remained significantly elevated. Further diagnostics revealed MTC in the left thyroid lobe with local and regional metastases [87]. Comparable phenomena were observed in men. For example, a 43-year-old man with moderate COVID-19 had a PCT level of 94 ng/mL (normal: 0.00–0.10 ng/mL) and a calcitonin level of 2120 pg/mL (normal: up to 12 pg/mL). Despite antibiotic therapy and clinical improvement, the high PCT level persisted. Further diagnostics confirmed the presence of MTC, and thyroidectomy with bilateral neck lymph node dissection significantly reduced PCT levels by the third postoperative day [88]. Similarly, in a 46-year-old man, low CRP levels but high PCT levels led to the diagnosis of MTC and, consequently, timely treatment [89]. These case analyses suggest that the widespread diagnostic use of CRP and PCT during COVID-19 to evaluate the efficacy of antibiotic therapy may have indirectly contributed to faster detection of MTC. The studies also highlight the necessity for careful management of treatment protocols for COVID-19, PCT testing, and TC to improve patient outcomes.

Epidemiological studies also indicate a relatively frequent occurrence of thyroid dysfunction in patients with COVID-19, often accompanied by non-thyroidal illness syndrome (NTIS) (26%) and thyrotoxicosis (10%) [90]. In a study involving 191 COVID-19 patients, thyroid dysfunction was observed in as many as 13.1% of cases. The authors suggested that COVID-19 could potentially exacerbate pre-existing autoimmune thyroid disease. Interestingly, this study showed that it was not TSH but rather fT3 that was associated with the severity of SARS-CoV-2 infection—lower levels of this hormone correlated with poorer prognosis [91]. In a similar study conducted among COVID-19 patients, an even higher incidence of thyroid dysfunction (25.4%) was observed, including thyrotoxicosis (20.2%) and hypothyroidism (5.2%). However, it was suggested that TSH, rather than fT3, had prognostic significance, as low TSH levels were associated with high levels of the inflammatory cytokine interleukin-6 (IL-6), suggesting that COVID-19 may be linked to a high risk of thyrotoxicosis [92].

There has even been a hypothesis proposing that, in the general population, thyroiditis related to COVID-19 may be widespread in the weeks and months following even mild COVID-19 infection [93]. This underscores the need not only to improve clinical management protocols but, more importantly, to take actions aimed at raising awareness within the community about the potential connections between COVID-19 and thyroid dysfunction.

### 3.2. Molecular Disorders Induced by SARS-CoV-2

The SARS-CoV-2 pandemic has demonstrated that recovering from COVID-19 can lead to both immediate and long-term complications, especially in individuals with pre-existing conditions. A significant number of people worldwide (over one million) have exhibited various atypical symptoms lasting more than three months after recovering from severe COVID-19. In response, the World Health Organization (WHO) has declared post-COVID-19 syndrome (PCS) as another epidemic of the 21st century [94]. PCS can cause long-lasting changes in single organs or multi-organ alterations, as evidenced by the presence of unusual proteins, such as neutrophil elastase and neuron-specific enolase, in the blood serum of patients with COVID-19-related complications [95,96]. Symptoms of PCS include difficulty concentrating, cognitive dysfunction, amnesia, depression, fatigue, and anxiety. Risk factors for persistent neuropsychiatric symptoms in PCS include advanced age, female sex, and the severity of pre-existing conditions such as diabetes. Diabetes in PCS is often linked to tachycardia, sarcopenia, microcirculatory dysfunction, or organ damage [97,98,99,100,101]. In some cases, PCS is caused by an immune response triggered by the SARS-CoV-2 infection [102]. However, the internal organ complications resulting from COVID-19 primarily stem from the virus’s intrinsic properties, specifically the presence of two key proteins: spike protein (S), which enables the virus to enter host cells, and SARS-CoV-2 main protease (TMPRSS2), which facilitates viral fusion with the host cell, transcription, and replication. Notably, TMPRSS2 lacks any homolog in human proteins [103].

SARS-CoV-2 enters cells by the spike protein’s recognition of the angiotensin-converting enzyme 2 (ACE2) receptor located on the cell membrane. ACE2 expression has been identified in the lung epithelium, blood vessels of the kidneys, cardiac muscle, and thyroid follicular cells. This explains the variety of pulmonary and extrapulmonary symptoms observed in COVID-19 [104].

The interaction of SARS-CoV-2 with host cell membrane receptors, its entry into cells, and intensive replication can disrupt multiple metabolic pathways, even in individuals without prior symptoms of metabolic or neurological diseases. This may be due to SARS-CoV-2 initiating mutations in genes encoding proteins essential for these biochemical pathways [105,106,107]. For instance, patients with COVID-19 who experienced hypoxia but had no diagnosed comorbidities and were hospitalized due to severe disease often exhibited significantly elevated serum glucose and potassium levels compared to patients with oxygen saturation levels > 95%. These findings suggest a mutagenic effect of SARS-CoV-2, specifically mutations in genes encoding the SUR1/KIR.2.6 potassium channel in the pancreas. Such mutations lead to membrane depolarization and inhibition of insulin secretion during hypoxia [105,108,109]. This hypothesis is supported by observations from other research groups, which have reported an increase in the incidence of diabetes associated with the absence of or significant reduction in insulin secretion in individuals who have recovered from COVID-19 [110]. It is known that reduced insulin secretion occurs not only in type 1 diabetes mellitus (T1DM) but also in maturity-onset diabetes of the young (MODY), which is molecularly linked to mutations in the SUR1/KIR.2.6 channel genes. MODY is often misdiagnosed as T1DM [111,112]. Early comparative analyses conducted worldwide have confirmed an increased incidence of autoimmune types of diabetes following COVID-19. For example, in Finland, the incidence of T1DM (and diabetic ketoacidosis requiring intensive care) increased from 2.89/100,000 person-years in 2016–2019 to 9.35/100,000 person-years in 2020 [113]. A meta-analysis involving 503 million cases from Europe and the United States also corroborated this observation [114]. Moreover, it has been shown that in individuals with pre-existing conditions such as type 2 diabetes mellitus (T2DM) or advanced diabetic nephropathy, COVID-19 can significantly elevate levels of intracellular proteins, such as neutrophil elastase (NE), neuron-specific enolase (NSE), and neuronal protein S100B. These proteins, which are not typically found in blood serum, indicate internal organ damage potentially linked to genetic mutations [115]. Since the thyroid gland, like the pancreas, contains receptors for SARS-CoV-2 (e.g., ACE2) and TMPRSS2 protein, it is likely that COVID-19 similarly disrupts its function at various molecular levels. Furthermore, the virus’s entry into thyroid cells may interfere with the hypothalamic–pituitary–thyroid axis.

### 3.3. The Impact of COVID-19 and PCS on Thyroid Dysfunction

**Figure 1:**
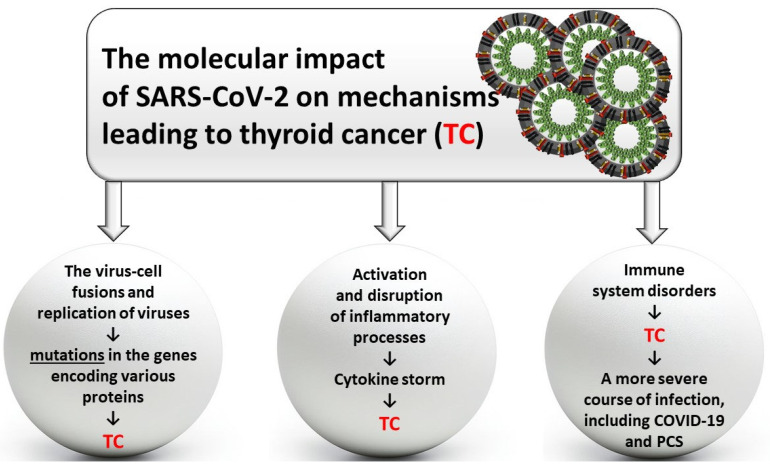
Molecular pathways leading to thyroid carcinogenesis potentially influenced by SARS-CoV-2.

The diagnosis of patients with COVID-19 and coexisting thyroid complications is multifaceted and further complicated by overlapping symptoms. Thyroiditis and changes in thyroid function can mimic or exacerbate COVID-19 symptoms, making diagnosis and prognosis more challenging [116]. Significant gaps in knowledge persist regarding thyroid complications in COVID-19 patients, particularly concerning the underlying mechanisms of these disorders. While registry analyses do not indicate increased mortality from thyroid cancer (TC) during the COVID-19 pandemic, the structure and functions of the thyroid gland suggest a likely impact of SARS-CoV-2 on the induction and progression of TC (Figure 1).

**Figure 2:**
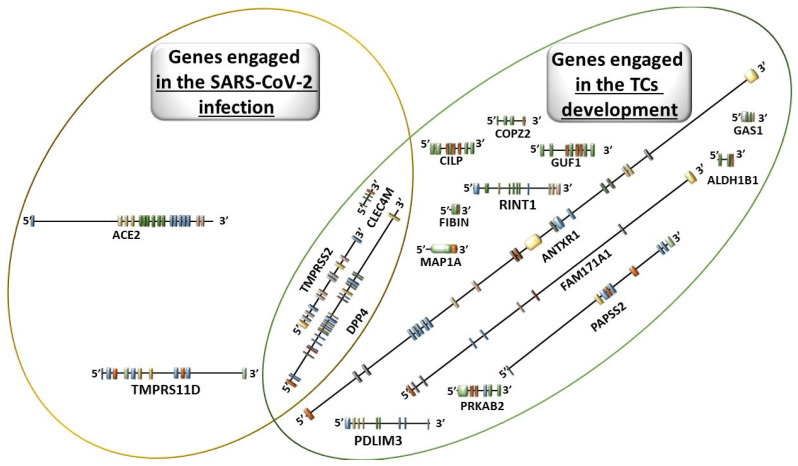
Genes engaged in the SARS-CoV-2 infection and engaged in the TCs development.

Due to the presence of genes encoding two key proteins for SARS-CoV-2, namely ACE2 and TMPRSS2, in thyroid cells, it is possible for this virus to be captured, fuse with the cell membrane, subsequently enter the cell, and replicate. It is also speculated that the frequent co-occurrence of cancers in COVID-19 patients may not be coincidental or merely age-related but rather associated with specific patterns of SARS-CoV-2 receptor expression in various organs, including the thyroid gland [117]. Molecular mechanisms induced by the interaction of SARS-CoV-2 with ACE2 show significant variations in the expression of different thyroid-related genes. Studies have demonstrated that thyroid-specific genes encoding proteins such as ACE2, TMPRSS11D, TMPRSS2, CLEC4M, and DPP4 may interact with the virus. Moreover, significant changes in the expression of *TMPRSS2*, *CLEC4M*, and *DPP4* genes have been observed in thyroid cancer (TC) tissues [118]. Hu et al. reported that tumor tissues in COVID-19 patients with TMPRSS2-ERG (T2E) alterations in metastatic TC may exhibit increased susceptibility to SARS-CoV-2 infection, thereby worsening prognosis [119]. This conclusion was derived from bioinformatics analysis and thus requires validation through clinical studies [119]. A summary of genes involved in thyroid cancer development and SARS-CoV-2 infection is graphically presented in Figure 2.

The presence of SARS-CoV-2 may not only induce mutations in various genes but also lead to a cytokine storm through other mechanisms, which can result in thyroid cancer (TC). The triggered inflammatory response in thyroid cells can not only disrupt the gland’s secretory function but also activate pro-oxidative mechanisms, immune system disturbances, and immunosuppression, which favor the development of neoplastic changes [120,121]. The thyroid gland plays a direct role in the body’s immune response to pathogens, as thyroid hormones act as modulators of this response. However, thyroid cancer cells can cause immune system dysfunction by weakening the cytotoxicity of T lymphocytes and reducing their proliferation [122]. The presence of a malignant tumor can complicate cytokine release, procoagulant processes, and endothelial dysfunction in patients. All of these factors can contribute to a more severe course of COVID-19 [80]. As a result, this increases the patient’s susceptibility to viral infections, such as those caused by SARS-CoV-2. It is currently known that SARS-CoV-2 infection can directly and indirectly affect the thyroid gland, even leading to apoptosis of cells [123,124]. Destruction of the gland may also occur indirectly through the induction of local and systemic inflammatory responses triggered by the virus—post-mortem studies of individuals suffering from COVID-19 have shown destruction of follicular and perifollicular thyroid cells [125], which may lead to thyroid cancer (TC). The replication of SARS-CoV-2 in thyroid cells often leads to thyroid damage and dysfunction (subacute inflammations, hypothyroidism, and autoimmune diseases), with symptoms such as back stiffness, neck pain, fever, muscle pain, lethargy, and an enlarged, inflamed thyroid gland [126]. The resulting damage is reinforced in cells due to the intensified release of pro-inflammatory cytokines (cytokine storm), triggered by the activation of genes encoding Janus kinase (JAK), signal transducer and activator of transcription (STAT), nuclear factor kappa-B (NF-κB), and mitogen-activated protein kinase (MAPK). Activation of these transcription factors increases the levels of interleukins (IL), interferons (IFN), tumor necrosis factors (TNF), and chemokines, promoting systemic inflammation and organ damage—a life-threatening condition [127]. Moreover, the cytokine storm in the acute phase of COVID-19 can lead to a systemic inflammatory response that indirectly affects thyroid function [128]. This condition can disrupt the hypothalamus–pituitary–thyroid axis, inducing changes in thyroid hormone production, potentially leading to transient hypothyroidism [129]. Additionally, thyroxine (one of the thyroid hormones) can increase the internalization of SARS-CoV-2 into cells, worsening the prognosis of COVID-19 [103]. SARS-CoV-2, like SARS-CoV-1 [130], damages thyroid follicular cells and can cause hypothyroidism. Furthermore, COVID-19 may induce thyroid disease in patients without prior history of thyroid disorders, such as sick euthyroid syndrome, Hashimoto’s thyroiditis (HT), and Graves’ disease (GD) [131]. It has also been observed that elevated IL-6 levels disrupt the production and functionality of thyroid-stimulating hormone (TSH), paving the way for the pathogenesis of thyrotoxicosis in COVID-19 [132]. Given the mechanisms indicating an increased risk of thyroid cancer (TC) in patients with COVID-19 and post-COVID-19 syndrome (PCS), further studies are needed to provide data to verify this association.

### 3.4. The Impact of Medications Used in the Treatment of Thyroid Diseases (Including Thyroid Cancer) in COVID-19

Individuals with oncological diseases are at an elevated risk of severe COVID-19 progression and higher mortality compared to the general population [133]. SARS-CoV-2 infection in patients with thyroid cancer (TC), compared to non-oncological patients, increases the risk of multi-organ damage. This is due to the development of a cytokine storm or immune system suppression, often caused by both the cancer itself and anti-cancer treatments such as chemotherapy or immunosuppression [103]. The severity of COVID-19 depends on the body’s inflammatory and immune response to the infection, which can particularly affect patients with TC. Immune dysfunction may arise from the cancer itself or as a side effect of treatments. Literature reports highlight cases where TC treatments did not worsen COVID-19 outcomes or increase mortality rates. For instance, studies have shown that TC patients can have a similar mortality rate compared to non-cancer groups, and treatment with radioactive iodine (RAI) and its cumulative dose does not negatively impact COVID-19 severity or mortality within the studied cohort [11]. As in the control group, factors such as age, diabetes, asthma/COPD, heart failure, chronic kidney disease, coronary artery disease history, use of RAS blockers, and low lymphocyte counts were associated with mortality in TC patients [11,82]. An elevated risk of COVID-19 mortality has been noted among TC patients with the following presentations: (1) well-differentiated but aggressive TC in advanced stages, with vascular invasion, lymph node metastases, or distant metastases; (2) poorly differentiated types, particularly medullary thyroid carcinoma (MTC) and anaplastic thyroid carcinoma (ATC); (3) patients without cancer but receiving high doses of levothyroxine (T4) as replacement therapy post-thyroidectomy [126,134]. Conversely, a lower COVID-19 mortality risk has been observed in patients with well-differentiated TC, such as follicular thyroid carcinoma (FTC) and papillary thyroid carcinoma (PTC) [126]. Furthermore, a multicenter report analysis indicated that among differentiated thyroid carcinoma (DTC) patients, advanced age and comorbidities are significant factors increasing the risk of severe COVID-19 progression [85]. It was also noted that a relatively higher incidence of COVID-19 occurred in TC patients treated with multikinase inhibitors (MKIs) [135]. Based on observations of treatment efficacy during COVID-19, it has been concluded that treatment should not be delayed in the following cases: (1) aggressive ATC or poorly differentiated TC without *BRAF^V600E^* mutation, or progressing (clinically aggressive) DTC and MTC; (2) suspected malignant thyroid tumors with documented progression; (3) large goiters causing significant airway compression and symptoms; (4) rapidly growing tumors; (5) suspected ATC [136,137].

Medications used in the treatment of thyroid disorders, including various types of thyroid cancer (TC), can influence the initiation and severity of COVID-19. They may also interact with other medications, such as those used to treat COVID-19, which can sometimes necessitate the temporary discontinuation of thyroid-related drugs during the course of the infection. The primary drugs used to manage thyroid disorders, including TC, include the following: thyroid hormone analogs (Levothyroxine (T4)), inflammasome inhibitors (colchicine), multikinase inhibitors (vandetanib, cabozantinib, sorafenib, lenvatinib, dabrafenib in combination with trametinib, selpercatinib, entrectinib, larotrectinib, vemurafenib, pazopanib, sunitinib, anlotinib, axitinib, dovitinib, and everolimus), humanized monoclonal antibodies (pembrolizumab, spartalizumab), and corticosteroids. The therapeutic uses of these drugs and their potential effects on the progression of COVID-19 are described in detail in Table 2.

The drugs presented in Table 2, used in the treatment of various types of thyroid cancer (TC), primarily serve to inhibit the expression of mutated genes (*ALK*, *BRAFV600E*, *c-MET*, *EGFR*, *FGFR1–4*, *mTOR*, *NTRK*, *PDGFR*, *RET*, *ROS1*, *VEGFR*, *VEGFR1–3*, and *VEGFR2/3*), block the interaction of key protein units, and demonstrate antiangiogenic effects (e.g., creatine kinase inhibitors). Consequently, they often also affect the reduction of cytokine storms and the normalization of immune profiles (reduction of conventional T cell proliferation and maintenance of growth and activity of regulatory T cells) [182,183]. As observed, the use of most of these drugs is not neutral with respect to SARS-CoV-2 infection. The largest group of drugs used in TC treatment are MKIs. They influence the course of COVID-19 by activating or inhibiting various molecular pathways. For example, they may interact with genes whose expression is induced by SARS-CoV-2 (sorafenib), inhibit viral replication (dabrafenib), strongly bind to SARS-CoV-2 proteins (selpercatinib), hinder virus attachment and replication by blocking the substrate-binding domain necessary for initiating apoptosis in infected cells (vemurafenib), or disrupt the binding of SARS-CoV-2 to immunoglobulins associated with the cell surface (pazopanib). Additionally, humanized monoclonal antibodies (pembrolizumab and spartalizumab) may normalize the immune phenotype and restore T cell functions [188]. Furthermore, colchicine-related drugs may affect the inhibition of inflammasome signaling, reducing cytokine production. However, some of the drugs used, such as levothyroxine (a T4 hormone analog), increase the likelihood of SARS-CoV-2 binding to host cells. Levothyroxine interacts with integrin αvβ3, which is responsible for the adhesion of various components to cells [134], and also regulates the expression of genes encoding components responsible for the cytokine storm. Since all patients undergo replacement therapy with T4 hormone after thyroidectomy, this drug is a potential factor increasing the risk of contracting COVID-19 and a more severe course of the disease [134]. However, opinions among scientists are divided; for example, Smulever suggests a positive effect of T4 on the immune system [136]. Perhaps the relationship between T4 and the risk of developing COVID-19 and PCS is dose-dependent, but this issue remains unresolved. A negative impact on the course of COVID-19 has also been observed with the use of corticosteroids, which are commonly used in patients with brain metastases or extensive lung metastases during I131 treatment. There is a suspicion that, similar to previous coronavirus pandemics (MERS-CoV and SARS), corticosteroids may be associated with worse outcomes in patients [80].

## 4. Conclusions

So far, genes have been identified that are responsible for the formation and development of various types of thyroid cancer (TC), ranging from benign to the most aggressive. It has been shown that mutations in the RAS family genes (*NRAS*, *KRAS*, *HRAS*) are most commonly associated with follicular thyroid carcinoma (FTC), but they are also present in three other types, including anaplastic thyroid cancer (ATC). Additionally, mutations in the *BRAF* gene are linked to papillary thyroid carcinoma (PTC) but can also occur in ATC. Mutations in some genes are relatively well understood, such as in the *TP53* gene, while the cellular consequences of some mutations are still poorly understood, such as in the *EIF1A* gene.

The limitations in access to diagnostics and treatment of TC caused by the COVID-19 pandemic did not lead to an increase in mortality due to TC. However, it has been shown that the diagnosis of individuals with COVID-19, especially measuring CRP and PCT, contributed in some cases to the early detection of TC—the observed high PCT level with a low CRP level was a signal suggesting a cause other than bacterial infection, most often TC (commonly medullary thyroid carcinoma, MTC), as PCT is a precursor of calcitonin, which is produced in excess by the thyroid in TC. The medications used by patients with TC were also not neutral to COVID-19. They showed antiviral activity, for example, inhibiting the replication of SARS-CoV-2 or reducing the cytokine storm, thereby alleviating the inflammatory and immune response. Further research on these drugs may lead to their approval as effective treatments against viruses as challenging as SARS-CoV-2. Additionally, although it has not yet been definitively proven that COVID-19 or post-COVID-19 syndrome (PCS) can cause thyroid carcinogenesis, due to the fact that thyroid cells have two key proteins, ACE2—the receptor for SARS-CoV-2, and TMPRSS2—a protease facilitating viral fusion with the cell, it is likely that having had COVID-19 or its long-term complications may contribute to the development of TC. COVID-19 may also trigger a cytokine storm, the consequence of which could be TC. High cytokine levels often disrupt the secretory function of the gland, activate pro-oxidative mechanisms, and lead to immune system dysfunction and immunosuppression—processes that favor the development of cancerous changes. It is therefore likely that COVID-19 and PCS could induce TC, but this hypothesis requires further confirmation.

## Figures and Tables

**Table 1:** Characteristics of genetic mutations appearing in thyroid neoplasms.

Protein That Is Mutated	Role of Protein Encoded by Gene	Oncogenes Diagnosed in Thyroid Cancers	Etiology of Gene Mutation	Consequences of the Mutation	Types of Thyroid Cancers with the Mutation (Frequency Within TC, %)	Kinds of Cancer with the Particular Mutation
BRAF	Encodes serine-threonine kinase of B-Raf proteins engaged in sending signals inside cells and in managing cell growth.	*BRAF^V600E^* *BRAF^K601E^*	The point mutation: valine (V) is substituted by glutamic acid (E) at amino acid 600 of B-RAF. The point mutation: lysine (L) is substituted by glutamic acid (E) at amino acid 601 of B-RAF.	Tumor driven by *BRAF^V600E^* induces mitogen-activated protein kinase MAPK-signaling (does not respond to the negative feedback from *ERK* to *RAF*). BRAF^V600E^ alters methylation (as a consequence of expression of numerous genes), promoting PTC tumorigenesis. Activation of the MAPK pathways.	PTC (80%), ATC FTC	Colorectal cancer [8,9] Thyroid adenoma [10]
RAS	GTPase is involved in regulating cell division, differentiation and apoptosis. They code p21 protein, engaged in cell growth, proliferation, and differentiation.	*NRAS* *HRAS* *KRAS*	Mutations occur in codons 12, 13 (*KRAS*), and 61 (*HRAS* and *NRAS*) of *RAS* gene.	Tumors driven by *RAS* decrease MAPK-signaling (responding to the negative ERK feedback). Translation of altered p21 protein—uncontrolled cell proliferation.	FTC (30–45%), PDTC (20–40%), ATC (10–20%), DTC, MTC	Lung, pancreatic, colorectal cancer [11]
EIF1A	Eukaryotic translation initiation factor 1A. It is a component of the 43S pre-initiation complex (PIC) required for the binding of the 43S complex to the 5′ end of capped RNA (controlling the initiation of protein synthesis). Encoded on human chromosomes X and Y (EIF1AX, EIF1AY).	*EIF1AX (EIF1AX-A113splice in TC)*	Mutations in exons numbers 2, 5 and 6.	*EIF1AX* mutations is highly associated with *RAS* mutations. The increase or altered function of proteins owing alteration for specific substitutions in the C- and N-terminal tails, e.g., the promotion of the G1/S phase transition through the transcriptional repression of p21. The cellular mechanisms caused by this mutation are poorly understood.	ATC (11–33%), PDTC (11%), FTC (2.5–7.4%), PTC (1–2.3%)	Uveal melanomas, breast, ovarian cancers, Benign thyroid adenomas [12,13,14,15,16]
PI_3_K	Phosphatidylinositol 3-kinase, the component of the PTEN/PI3K/AKT pathway, regulates cell cycle progression, cell survival, adhesion, motility and spreading, angiogenesis, glucose homeostasis, and cell size and organ size control.	*PIK_3_CA*	Mutation in exons numbers 9 and 20; amplification of catalytic subunit of p110alpha of *PI_3_K.*	Activation of the PTEN/PI3K/AKT pathway.	ATC (23%), FTC (8%), PTC (1%)	Colorectal, gastric, breast, ovarian cancers, and high-grade brain tumors [17,18]
PTEN	The phosphatase and tensin homolog, a key negative regulator of the PI_3_K/mTOR pathway.	*PTEN/PHTS*	The inactivation of the *PTEN* gene by epigenetic mutation caused by aberrant methylation or gene deletion.	Leads to a cluster of tissue overgrowth syndromes termed PTEN hamartoma tumor syndrome (PHTS).	PTC (1.5–5%), FTC (1–4.5%)	Breast, ovarian, endometrial, colon, prostate cancer, and glioblastomas [17,19]
DICER1	Ribonuclease III involved in cleaving double stranded pre-microRNAs into mature miRNAs.	*DICER1*	This mutation affects the metal ion-binding capacity of the RNAse domain, interfering with the catalytic site of the enzyme, reducing the production of 5p miRNAs.	Changes in post-transcriptional regulation of gene expression. Germline mutations have been associated with a familial tumor susceptibility syndrome.	FTC (7–10%)	Lungs, kidneys, ovaries cancers, multinodular goiter (MNG), Sertoli–Leydig cell tumors (SLCT), cystic nephroma, pleuropulmonary blastoma, cancer of cervical embryonal, rhabdomyosarcoma, and Wilms tumor [20]
EZH1	Enhancer of zeste homolog 1—a member of the Polycomb group protein complex, components for prevention of cancer stem cell development.	*EZH1^Q571R^* *EZH1^Y642F^* *EZH1^M349L^*	The point mutation called hot-spot mutation (c.1712A>G; p.Gln571Arg) in the enhancer of zeste homolog 1.	*EZH1* are associated with alterations in cAMP pathway genes.	FTC: 3–5.9%	Uveal melanoma [21]
SPOP	Speckle-Type Poz Protein, an E3 ubiquitin ligase adaptor protein. SPOP interacts with CUL3 during ubiquitination of substrates.	*SPOP^SCR3^* *SPOP^AR^* *SPOP^ERA^*	Missense mutations, loss of expression.	SPOP mutants promote the error-prone non-homologous end joining (NHEJ) pathway.	PTC:	Prostate, liver, and endometrium cancer [22,23]
p53	p53 drives DNA repair, arrests, cell-cycle, senescence, and apoptosis when it is phosphorylated by DNA damage response (DDR) kinases. It acts a key failsafe mechanism of cellular anti-cancer defenses.	*TP53*	Mutations in 5–9 exons of gene encoding Tp53.	Disorders in mechanisms of cellular anti-cancer defenses.	ATC: 58–80%, PDTC: 26% FTC: 11% PTC: 3.5%	Breast, brain, adreno cortical cancers, central nervous system tumors, and sarcomas [24,25,26]
TERT	Encodes the reverse transcriptase (subunit of the telomerase complex), elongating the telomere portion of chromosomes (adds repeated sequences). Its expression and activity are strongly increased in cancer cells (usually is absent or low).	*TERT^C228T^* *TERT^C250T^*	Point mutations.	Ensures chromosomal stability (maintains telomere length), leads cancer cells to senescence.	PTC: 7–22% FTC: 14–17% DTC: 10% ATC: 70%, PDTC: 40%	Various types of cancer [25,27,28]
FGF	fibroblast growth factor					
	receptors for TSH					
Chromosomal Rearragments						
RET	Rearranged during Transfection (RET)— the tyrosine kinase, is composed by an extracellular (EC), a transmembrane (TM), and an intracellular (IC) portion. The functional tyrosine-kinase receptor (RTK) of glial cell line-derived neurotrophic factor (GDNF), neurturin (NRT), artemin (ART), and persephin (PSF) growth factors. These growth factors bind to GFRs (GDNF family receptor), forming a complex that mediates RET dimerization and activation.	*ACBD5-RET*, *AFAP1L2-RET*, *AKAP13-RET*, *ANKRD26-RET*, *RET/PTC1*, *DLG5-RET*, *ERC1-RET*, *FKBP15-RET*, *RET/PTC5*, *HOOK3-RET*, *KIAA1468-RET*, *RET/PTC8*, *RET/PTC3*, *PCM1-RET*, *RET/PTC2*, *RUFY2-RET*, *SPECC1L-RET*, *TBL1XR1-RET*, *SQSTM1-RET*, *RET/PTC6*, *TRIM27-RET*, *RET/PTC7*, *UEVLD-RET*, *TFG-RET*, *RET/PTC1*, *RET/PTC3*, *PPFIBP2-RET*, *MYH13-RET*	Gene amplification, fusion, single base substitutions (or small insertions), deletions either in *RET* sequences.	It triggers signaling along the MAPK pathway, leading to an uncontrolled proliferation. It activates the transcription of the *RET* tyrosine-kinase domain.	PTC PDTC MTC	Lymphoma, cholangiocarcinoma, lung adenocarcinoma, breast invasive carcinoma, spindle cell tumor of soft tissues, colorectal carcinoma, stomach adenocarcinoma, spitzoid neoplasms, invasive mucinous lung adenocarcinoma, infantile myofibromatosis, intraductal carcinoma of the salivary gland, lipofibromatosis, and lipofibromatosis-like neuronal tumors [29,30]
PAX8 PPARγ	PAX8—one of the paired box transcription factor, necessary for thyroid development, drives the expression of genes encoding thyroglobulin, thyroid peroxidase, and the sodium iodide symporter. PPARγ, the nuclear receptor of transcription factors, regulates adipogenesis and modulates lipid metabolism and insulin sensitivity. PPARγ has anti-inflammatory activity (a tumor suppressor).	*PAX8/PPARG*	The *PAX8/PPARG* rearrangement— a translocation between chromosomal regions 2q13 and 3p25.60—results in a fusion transcript: the sequence of PAX8 (2q13) is fused in frame with the exons of *PPARγ1* (3p25). The PAX8 promoter (highly active in thyroid follicular cells) drives the expression of the fusion transcript of *PPFP.*	Production of fusion protein PAX8/PPARγ (PPFG). PPFG acts as dominant negative inhibitor of wild-type PPARγ and/or as a unique transcriptional activator of subsets of PPARγ and PAX8-responsive genes.	FTC (30–35%) PTC (17.6%)	Non-invasive follicular thyroid neoplasm with papillary-like nuclear features [31,32,33,34,35]

**Table 2:** The impact of medications used in patients with thyroid dysfunction (including TC) on COVID-19.

Medication	The Impact of the Drug on the Course of COVID-19
Levothyroxine (T4) (Euthyrox, Letrox and others)	The drug is routinely used in patients with postoperative hypothyroidism. Similar to endogenous thyroid hormones, T4 stimulates the immune system to defend against viruses (T-cell activation, secretion of IFN-γ and cytokines, regulation of chemotaxis, and phagocytosis) [105]. T4 is believed to influence the fusion of SARS-CoV-2 with target cells and activate human platelets. Consequently, patients taking high doses of T4 are at a higher risk of COVID-19 infection and have a poorer prognosis, including potential thrombotic complications [134].
Colchicine	Colchicine is an anti-inflammatory drug used to treat various conditions, including gout and recurrent pericarditis; it reduces neutrophil chemotaxis, inhibits inflammasome signaling, and decreases cytokine production. It has been reported to lower mortality risk and improve clinical outcomes in COVID-19 patients. Zhang identifies colchicine as an inhibitor of thyroid cancer [138,139,140].
Vandetanib	Belonging to the multikinase inhibitors, Vandetanib is an FDA-approved drug for the treatment of advanced medullary thyroid cancer (MTC). It selectively blocks the epidermal growth factor receptor (*EGFR*), vascular endothelial growth factor receptors (*VEGFR2/3*), and *RET* tyrosine kinase. Like other multikinase inhibitors (MKIs), it suppresses cytokine secretion, making it potentially useful in the treatment of COVID-19. Studies on animal models and cell cultures have demonstrated that Vandetanib significantly reduces inflammatory cytokine levels and immune cell infiltration in the lungs, suggesting its potential application in COVID-19 treatment [141].
Cabozantinib	Cabozantinib belongs to the class of multikinase inhibitors. It is aimed at inhibiting *VEGFR*, *c-MET*, and *RET*; it is an FDA-approved drug for the treatment of progressive and symptomatic MTC and has a strong antiangiogenic effect [142]. Although cabozantinib exhibits strong anti-inflammatory activity, caution is suggested when using it for the treatment of COVID-19 or PCS [143].
Sorafenib	Sorafenib belongs to the class of multikinase inhibitors. It is targeted at inhibiting *VEGFR1–3*, *PDGFR*, and *RET*. It is approved for the treatment of RAI-resistant DTC, with proven efficacy in treating metastatic thyroid cancer, including PTC and RAI-resistant DTC [144,145,146], as well as in the treatment of MTC [147]. However, its efficacy in treating ATC is debated [148,149]. Sorafenib has been shown to exhibit antiviral activity. Additionally, it interacts with genes whose expression is induced by SARS-CoV-2, making it a potential candidate for the treatment of COVID-19 [150]. However, it may interact with other drugs used in COVID-19, and its use should be immediately discontinued in cases of hypokalemia and fever [151].
Lenwatynib Dabrafenib in combination with trametinib	Lenvatinib, like other MKIs, targets tumor angiogenesis [152]. It is aimed at inhibiting *VEGFR1–3*, *FGFR1–4*, *PDGFR*, and *RET*. Single patient studies involving DTC and COVID-19 show no contraindications for continuing treatment despite viral infection [151]. Both drugs have been approved by the FDA for the treatment of ATC with the *BRAF^V600E^* gene mutation (dabrafenib is a selective inhibitor of the BRAFV600E mutant kinase and belongs to MKIs, while trametinib is a mitogen-activated protein kinase inhibitor) [153]. Computational analysis has shown that dabrafenib exhibits antiviral properties against SARS-CoV-2 [154], and this suggestion was confirmed in cell studies—dabrafenib inhibited SARS-CoV-2 replication [155]. There are also studies suggesting the effectiveness of trametinib in treating COVID-19 [156].
Selperkatynib	Selpercatinib (a selective *RET* receptor inhibitor) belongs to the MKIs and has been approved by the FDA for the treatment of MTC with RET mutations [157,158]. Computational studies with elements of quantum mechanics suggest that this drug may strongly bind to SARS-CoV-2 proteins, thereby mimicking their functions, which could have therapeutic significance [159].
Entrectinib	Entrectinib belongs to the MKIs, specifically to the new inhibitors of anaplastic lymphoma kinase, c-ros oncogene 1 (ROS1) kinase. Bioinformatics analysis indicates the potential significance of entrectinib in the treatment of COVID-19 [160,161]. Additionally, entrectinib has shown antiviral activity against SARS-CoV-2 in human lung tissue [162].
Larotrectinib	Larotrectinib is a selective *NTRK* inhibitor belonging to the MKIs, approved by the FDA for the treatment of solid tumors with NTRK gene fusions [163]. Its efficacy has been demonstrated in the treatment of PTC [164]. Similar to entrectinib, its potential properties against COVID-19 have also been shown [165].
Vemurafenib	Vemurafenib is an MKI, a selective inhibitor of the mutated serine-threonine kinase *BRAF*, and strongly inhibits *ERK* phosphorylation [166]. In silico analysis showed that this drug inhibits virus binding and replication by blocking the substrate-binding domain (which triggers apoptosis in infected cells), making it a candidate for COVID-19 treatment [167].
Pazopanib, sunitynib, anlotinib, aksytynib i dovitinib	These are MKIs belonging to antiangiogenic creatine kinase inhibitors, showing efficacy in the treatment of thyroid cancer (TC) by inhibiting *VEGFR1–3*, *PDGFR*, *FGFR*, and *RET* [168]. The efficacy of pazopanib has been demonstrated in the treatment of patients with DTC or MTC [169,170,171], anlotinib in the treatment of MTC, even with metastases [172,173], and axitinib shows significant anticancer activity in all histological subtypes of advanced TC [174]. Dovitinib shows potential therapeutic significance in the treatment of locally advanced or metastatic DTC and MTC [175]. Given that these inhibitors clearly influence the reduction of Ebola virus infectivity and hepatitis C inflammation, it is possible that they may also affect SARS-CoV-2, as confirmed by studies [176,177]. It is most likely that pazopanib disrupts the binding of SARS-CoV-2 to immunoglobulins associated with the cell surface, which may make it effective in the treatment of COVID-19 [167].
Everolimus	Everolimus belongs to the MKIs and is an inhibitor of the serine-threonine kinase signaling pathway mTOR [178]. It has been approved by the FDA for the treatment of HER2(-) breast cancer and pancreatic neuroendocrine tumors [179]. Phase II studies have confirmed its therapeutic significance in locally advanced or metastatic thyroid cancer (TC) [180], such as in patients with DTC, MTC, and ATC [181]. It is hypothesized that everolimus, by inhibiting mTOR, may be effective in the treatment of COVID-19 by reducing conventional T cell proliferation, decreasing the cytokine storm, and maintaining the growth and activity of regulatory T cells [182,183].
Pembrolizumab Spartalizumab	Pembrolizumab is a humanized monoclonal antibody (anti-PD-1) approved by the FDA for the treatment of advanced melanoma and non-small cell lung cancer [184]. Clinical studies have shown that pembrolizumab also has anticancer effects in several patients with advanced DTC [185]. Spartalizumab is a humanized monoclonal immunoglobulin-4 antibody that blocks interactions with PD-L1 and PD-L2 [186], and its therapeutic effects have been demonstrated in ATC [187]. Analysis of the immune profiles of COVID-19 survivors showed that PD-1 blockade normalizes the immune phenotype and restores T cell functions, making pembrolizumab and spartalizumab effective in the treatment of COVID-19 [188].
Corticosteroids	Corticosteroids are commonly used in patients with brain metastases or extensive lung metastases during I^131^ treatment; there is a suspicion that, similar to previous coronavirus pandemics (MERS-CoV and SARS), corticosteroids may be associated with worse outcomes in patients [80].

## Abbreviations

ACE2—Angiotensin-Converting Enzyme 2; ACTH—Adrenocorticotropic Hormone; AKT: Protein Kinase B; ALDH1B1—Aldehyde Dehydrogenase 1 Family Member B1; ANTXR1—Anthrax Toxin Receptor 1; ART—Artemin; ATC—Anaplastic Thyroid Cancer; BRAF—B-Raf Proto-Oncogene, Serine/Threonine Kinase; BTN-PH—Benign Tubercle with Papillary Hyperplasia; CatL—Cysteine Protease L; CEA—Carcinoembryonic Antigen; CGRP—Calcitonin-Gene Related Peptide; CLEC4M—C-Type Lectin Domain Family 4 Member M; CILP—Cartilage Intermediate Layer Protein; CMV—Cribriform-Morular Variant; COPZ2—Coatomer Subunit Zeta 2; COVID-19—Coronavirus Disease 2019; DPP4—Dipeptidyl Peptidase 4; DTC—Differentiated Thyroid Cancer; EGFR—Epidermal Growth Factor Receptor; EIF1A—Eukaryotic Translation Initiation Factor 1A; EIF1AX—X variant of EIF1A; ERK—Extracellular Signal-Regulated Kinase; EZH1—Enhancer of Zeste Homolog 1; FAM171A1—Family With Sequence Similarity 171 Member A1; FIBIN—Fin Bud Initiation Factor Homolog; FNAs—Fine Needle Aspiration Biopsy; FTC—Follicular Thyroid Cancer; GAS1—Growth Arrest Specific 1; GUF1—GTPase Elongation Factor Homolog; IFN-γ—Interferon Gamma; MAP1A—Microtubule-Associated Protein 1A; JAK—Janus Kinases; MEN2—Multiple Endocrine Neoplasia Type 2; MERS-CoV—Middle East Respiratory Syndrome Coronavirus; MNG—Multinodular Goiter; MTC—Medullary Thyroid Cancer; PAPSS2—3′-Phosphoadenosine 5′-Phosphosulfate Synthase 2; NF-κB—Nuclear Factor Kappa-Light-Chain-Enhancer of Activated B Cells; PDTC—Poorly Differentiated Thyroid Cancer; PRKAB2—Protein Kinase AMP-Activated Non-Catalytic Subunit Beta 2; PCS—post-COVID-19 syndrome; PCT—procalcitonin, PDGFRA—Platelet-Derived Growth Factor Receptor Alpha; PDLIM3—PDZ and LIM Domain Protein 3; PI3K—Phosphoinositide 3-Kinase; PSF—Persephin; RAI—Radioactive Iodine; PTC—Papillary Thyroid Cancer; RAS—Rat Sarcoma Virus Oncogene; SARS-CoV-2—Severe Acute Respiratory Syndrome Coronavirus; SLCT—Sertoli-Leydig Cell Tumors; STAT—Signal Transducer and Activators of Transcription; TERT—Telomerase Reverse Transcriptase; TC—Thyroid Cancer; TMPRSS2—Transmembrane Serine Protease 2; TMPRS11D—Transmembrane Serine Protease 11D; TP53—Tumor Protein P53; VIP—Vasoactive Intestinal Peptide.
